# Trajectories of adjustment disorder symptoms in post-treatment breast cancer survivors

**DOI:** 10.1007/s00520-022-06806-z

**Published:** 2022-01-12

**Authors:** Lonneke M. A. Wijnhoven, José A. E. Custers, Linda Kwakkenbos, Judith B. Prins

**Affiliations:** 1 Department of Medical Psychology 926, Radboud University Medical Center, Radboud Institute for Health Sciences, PO Box 9101, 6500 HB Nijmegen, The Netherlands; 2 Clinical Psychology, Behavioural Science Institute, Radboud University, Nijmegen, The Netherlands

**Keywords:** Adjustment disorder, Breast cancer survivors, Trajectory analysis, Oncology, Psychology

## Abstract

**Objective:**

Breast cancer survivors (BCS) may experience problems to adjust to their situation after cancer treatment completion. In case of severe distress, an adjustment disorder (AD) might develop. This study investigates the course of AD symptoms during 1 year and its predictors in BCS up to 5 years post-treatment.

**Methods:**

BCS completed the Hospital Anxiety and Depression Scale (HADS) at baseline, 3, 6, and 12 months. HADS total scores were defined as no mental disorder (MD) symptoms (≤ 10), AD symptoms (11–14), and any other MD symptoms (≥ 15). Over the course of four assessments, symptom trajectories were a priori defined as no MD symptoms, AD symptoms, fluctuating AD symptoms below and above cut-offs, or any other MD symptoms. Complementary, latent class growth analysis (LCGA) was used to identify data-driven trajectories.

**Results:**

Among 293 BCS with complete data, the majority was classified as no MD symptoms (54.4%), followed by 37.5% in the fluctuating AD symptoms trajectory. Only 1.4% had AD symptoms, and 6.8% had any other MD symptoms. With LCGA (*N* = 459), three trajectories were found: stable no MD symptoms (58.6%), stable AD symptoms (32.9%), and high increasing any other MD symptoms (8.5%). Compared to BCS with no MD symptoms, BCS with fluctuating AD symptoms or any other MD symptoms were younger, less able to handle daily activities, and showed more social support discrepancy, neuroticism, and less optimism.

**Conclusions:**

Results of our study showed that AD symptoms in BCS up to 5 years post-treatment fluctuate over 1 year. It is thus important to appropriately assess AD over the course of 5 years post-treatment as AD symptoms can fluctuate.

## Background

Breast cancer is the most prevalent cancer type in women. Improved methods for early cancer detection and innovations in cancer treatment have increased the 5-year survival rate in breast cancer survivors (BCS), which is currently 91% in The Netherlands [2, 32]. As a consequence, more BCS are dealing with the long-term complications of their cancer treatment, including the psychological burden [5, 14, 24, 38]. When cancer-related distress is severe, an adjustment disorder (AD) can be diagnosed [10, 39], if symptoms are not solely an exacerbation of a pre-existing mental disorder (MD) and the criteria of another MD are not met. In The Netherlands, reimbursement of psychological interventions for cancer survivors is available if a MD such as an anxiety disorder or major depression is diagnosed. It is currently being investigated whether AD can be added to the reimbursement scheme.

In the DSM-V, AD has been defined as the presence of emotional and behavioural symptoms in response to an identifiable stressor(s) occurring within 3 months of the onset of the stressor(s). The accompanied distress is out of proportion to normal reactions to the stressor in social or cultural context. After ceasing of the stressor or its consequences, symptoms of AD resolve within 6 months (Criterion E); however, if stressors or its consequences continue, this may result in persistent AD [3]. After curative cancer treatment, continuous confrontation with stressors is possible due to for instance ongoing adjuvant endocrine therapies, imaging, and follow-up appointments as well as long-term consequences of cancer such as fatigue, fear of cancer recurrence, and reduced ability to work. Critics debate that a MD diagnosis based on distress symptoms alone medicalizes problems of living [6] and that the AD diagnosis is unclear in discriminating a MD from a normal stress reaction [10]. More insight in AD in relation to trajectories of psychological adjustment after cancer is necessary.

Predictors of AD related to cancer have not been thoroughly investigated [9]. In a large mixed cancer sample (*N* = 2141), higher education, having metastases, and being female were identified as predictors for AD [19]. In another study in cancer patients, the more commonly investigated symptom distress was found to be predicted by more neuroticism, and findings on optimism were inconclusive [12].

Among patients with breast cancer, the prevalence of AD was estimated to be 7.1% in the acute phase of treatment [30], 38.6% in the first year post-diagnosis [41], 14.4% in BCS [29], and 20% in BCS with a first recurrence of breast cancer [23] when assessed with (semi)structured interviews, the golden standard to diagnose AD in clinical settings [31]. It is advised to screen patients with cancer for psychosocial problems prior to conducting clinical assessments [34]. Although screening is common for depression [7], anxiety [20], and posttraumatic stress disorder [4], measures focussing on AD, e.g. Adjustment Disorder New Module [28], the Diagnostic Interview Adjustment Disorder [13], and International Adjustment Disorder Questionnaire [36], are mostly used for research purposes. The Hospital Anxiety and Depression Scale (HADS), a commonly used screening questionnaire in cancer survivors, measures emotional distress with symptoms of depression and/or anxiety [43]. Since these symptoms are in line with the diagnostic criteria of AD, the HADS might also be used to screen for AD symptoms. Several studies have reported that the HADS is sensitive to identifying cases of AD in patients with cancer [1, 25, 33].

Understanding the course of AD symptoms over time may help identify BCS who develop persistent AD. Distinct distress trajectories in patients with breast cancer were observed up to 8 months post-diagnosis [18, 22, 26, 27]. Only one study followed BCS up to 4 years [17]. Distress trajectories were identified as stable low (36–80%), stable high (9–15.4%) [17, 18, 22, 26, 27], recovery (5.6% and 12%) [26, 27], delayed recovery (7–27%) [17, 22, 26], and worsening (4.5% and 7.9%) [22, 27]. One study identified distress trajectories during active treatment (33.3%) and during the re-entry and survivorship phase (15.2%) [18]. Predictors that distinguished trajectories were age [26], physical symptoms [26, 27] at treatment completion [22], satisfaction with medical consultation [26], history of psychiatric illness [27], personal [17] and social resources [17, 27], mastery [18, 27], optimism [18, 26], neuroticism [18], and benefit finding [27]. Most studies [17, 18, 22, 26] used a growth mixture modelling approach to determine trajectories from a data-driven point of view, and one study used cut-off scores from a clinical point of view [27].

The primary aim of this study was to detect trajectories of AD symptoms during 1 year using the HADS in BCS, using both clinically relevant cut-off scores and a data-driven growth modelling approach. The secondary aim was to identify predictors for distinct trajectories.

## Methods

### Participants and procedure

Physicians invited 1205 BCS from three hospitals in The Netherlands to participate with an information letter. Eligible participants were cancer-free, ≥ 18 years old, with stages I–III breast cancer treated with curative intent, who finished primary cancer treatment in the past 5 years, and were able to complete questionnaires in Dutch. BCS currently on hormonal therapy or treatment with specific antibodies (trastuzumab) were also eligible. After informed consent, participants received a questionnaire booklet (paper-and-pencil) or email with a link to a secured online system. Questionnaires were sent upon enrolment and after 3, 6, and 12 months.

### Demographic, clinical, and psychosocial measures

Participants completed socio-demographic variables (e.g. age and employment status) and self-reported clinical variables (e.g. type of treatment and time since diagnosis) in the baseline questionnaire.

*AD symptoms* were assessed using the HADS, a 14-item questionnaire with subscales Anxiety (HADS-A, 7 items) and Depression (HADS-D, 7 items) [37]. Items are scored on a scale (range 0–3), resulting in subscale (0–21) and HADS total (0–42) scores. For cancer survivors [43], cut-off scores were identified of HADS total ≥ 10 or 11 for screening for MDs (sensitivity 0.80; specificity 0.74) and ≥ 15 for screening for depression (sensitivity 0.87; specificity 0.88).

*Social support* was measured with the Social Support List‐Discrepancies (SSL-D), a 34-item questionnaire (4-point Likert scale). The SSL-D measures the perceived discrepancy between the amount of received social support and the desired amount of social support [42], further referred to as social support discrepancy. A higher total score of SSL-D (range 34–102) indicates more social support discrepancy. The test–retest reliability is 0.85 and a Cronbach’s alpha of 0.95 [42].

*Optimism* was measured with the Life Orientation Test (LOT), a 12-item questionnaire (5-point Likert scale, 0 = “strongly disagree” to 4 = “strongly agree”) reflecting generalized optimism versus pessimism. Higher total scores (range 0–32, 4 filler items excluded) indicated more optimism. LOT-total Cronbach’s alpha is 0.76, and test–retest reliability is 0.79 [35].

*Neuroticism* was measured using the Big Five Inventory (BFI) [16], a 44-item questionnaire designed to measure the Big Five factor structure of personality (5-point Likert scale). The Neuroticism subscale (BFI-N) measures the trait of neuroticism opposed to emotional stability, with increasing scores indicating a larger tendency to experience negative emotions. This version of the BFI has a Cronbach’s alpha of 0.86.

### AD symptoms and trajectories

Our definition of “AD symptoms” was theoretically derived from the DSM-V definition of AD, which describes that AD symptoms are characterized by marked distress, while the distress should not meet criteria for another MD. Cut-off thresholds for the HADS have been established that are sensitive to detect any MD (score ≥ 11) and depression (score ≥ 15)[43]. As such, we have assumed that a HADS score of 11 to 14 (i.e. marked distress but not depression) are indicative of AD symptoms. This is in line with previous studies reporting that the optimal HADS total score for screening for AD is 10 or 11 [1, 25, 33]. Thus, we predefined categories on HADS total: (1) ≤ 10 was defined as “no MD symptoms”, (2) 11 to 14 as “AD symptoms”, and (3) ≥ 15 as “any other MD symptoms”. Trajectories were created based on HADS total over four assessments and defined as (a) no MD symptoms at all four assessments, (b) AD symptoms at all four assessments, (c) any other MD symptoms at all four assessments, and (d) fluctuating AD symptoms, i.e. an increase, decrease, or irregular pattern of HADS total.

### Data processing and statistical analyses

Missing item scores on the HADS were replaced by the participants’ subscale mean if at least four subscale items were answered [8]. Participants who completed the HADS all four assessments were considered completers, and participants who had a missing HADS or did not report date of birth or time since diagnosis were considered non-completers. Completers and non-completers were compared on demographic and clinical variables using t-tests for continuous variables and chi-square tests for categorical variables. Completers were assigned into our a priori defined trajectories based on their score above or below cut-off scores, and trajectories were compared on demographic and psychosocial variables with univariate testing (one-way ANOVA, chi-square tests, and post hoc analysis). Variables that were significantly associated with trajectories membership were entered in a final multinominal regression analysis. Analyses were performed with SPSS version 25.

Latent class growth analysis (LCGA) was conducted using MPlus version 7 to identify data-based trajectories (classes) over time for HADS total, following the guidelines described by Jung and Wickrama [21]. By estimating individual differences (variability) in parameters reflecting participants’ change in outcome over time, individuals are classified into latent classes based upon similar patterns in the outcome of interest (HADS). MPlus’ full information maximum likelihood estimation for handling missing data was applied.

Following the guidelines, a single-class growth curve model was specified, as well as a three-class model. To determine the number of classes, the three-class model was compared with a two-class and four-class model, and the four-class model was compared with a three-class and five-class model. In total, the fit of five unconditional latent class models (i.e. models with no covariates) were estimated, with one to five linear classes. The number of classes was determined based on fit indices, model parsimony, and clinical interpretability. The model with the best fit has the smallest Bayesian Information Criterion (BIC) and significant *p*-values (*p* < 0.05) for the Vuong-Lo-Mendell Rubin Likelihood Ratio Test (LMR-LRT) and the Bootstrap Likelihood Ratio Test (BLRT), which indicate that a model with a *k* number of classes has a better fit than a model with k-1 number of classes. Other considerations were a higher entropy statistic (near 1.0), indicating the degree to which latent trajectories may be clearly distinguished, and higher posterior probabilities of group membership (near 1.0), indicating the degree to which individuals have been correctly classified into a class. For clinical interpretability, we also considered the number of participants (not less than 5% of total sample (*n* ≥ 23)) of the identified classes. For each individual patient in the database, the predicted class of the best fitting model (i.e., with the optimal number of subgroups) was obtained.

## Results

### Sample characteristics

Of the 1205 eligible BCS who were invited, 459 participants (38.1%) consented and completed the HADS at least once. Demographic and clinical variables of completers, non-completers, and the full sample are shown in Table 1. Compared to non-completers, completers were older (*p* = 0.002) and had a lower education level (*p* = 0.026).

**Table 1:** Socio-demographic and medical characteristics of participants

		**Completers** *N* = 293 (valid %)	**Non-completers** *N* = 166 (valid %)	**Full study sample** *N* = 459 (valid %)
Dutch nationality		287 (98.0%)	162 (98.8%)	449 (99.3%)
Age (mean, years (SD^a^; range))		57.8 (9.3; 33.0–87.6)	54.8 (10.0; 33.2–83.8)	56.7 (9.7; 33.0–87.6)
Marital status		228 (78.4%)	138 (83.6%)	366 (80.3%)
Children		240 (82.5%)	143 (86.7%)	383 (84.0%)
Education	Primary	65 (22.6%)	24 (14.7%)	89 (16.0%)
	Secondary	146 (50.7%)	78 (47.9%)	224 (49.7%)
	Tertiary	77 (26.7%)	61 (37.4%)	138 (30.6%)
Time since diagnosis (mean, months)		33.1 (SD 16.1)	33.1 (SD 16.1)	33.3 (SD 16.0)
Time since end of treatment (mean, months)		26.8 (SD 16.6)	28.7 (SD 16.8)	28.6 (SD 16.7)
Breast saving surgery		189 (64.5%)	98 (60.1%)	287 (62.9%)
Ablatio		39 (13.3%)	19 (11.7%)	58 (12.7%)
Breast amputation		75 (25.6%)	55 (33.7%)	130 (28.5%)
Chemotherapy		206 (70.3%)	123 (75.0%)	329 (72.0%)
Radiotherapy		226 (77.4%)	121 (73.8%)	347 (76.1%)
Hormone therapy		193 (65.9%)	102 (62.2%)	295 (64.6%)
Trastuzumab/Herceptin		37 (12.7%)	24 (14.6%)	61 (13.4%)

^a^Standard deviation

### Trajectories based on cut-off scores

At group level, the average of all four HADS-assessments was 72.1% with no MD symptoms, 12.9% AD symptoms, and 15.0% any other MD symptoms. Classification in trajectories resulted in 157 BCS (53.6%) in the trajectory no MD symptoms, 4 BCS (1.4%) in the trajectory AD symptoms, 20 BCS (6.8%) in the trajectory any other MD symptoms, and 112 BCS (38.2%) in the trajectory fluctuating AD symptoms.

### Predictors of trajectories based on cut-off scores

Given the low number of BCS in the trajectory AD symptoms, these BCS were merged with the trajectory fluctuating AD symptoms (stable&fluc-AD symptoms) and compared to the trajectory no MD symptoms and trajectory any other MD symptoms on demographic and psychosocial characteristics at baseline (Table 2).

**Table 2:** Trajectories based on cut-off scores: characteristics and multinominal regression analysis (Stable&fluctuating AD symptoms and any other MD symptoms versus no MD symptoms)

		**No MD symptoms**			**Stable&fluctuating AD symptoms**				**Any other MD symptoms**			
**Predictor**			*Χ*^2^	*p*		*B*	Wald	Exp (B) (95% CI)		*B*	Wald	Exp (B) (95% CI)
Intercept		n.a	7.2	.028	n.a	− 3.17	2.05	n.a	n.a	− 10.75	6.56**	n.a
Age (mean years (SD^a^; range))		58.9 (8.5; 37–87)	7.4	.025	56.1 (9.7, 33–78)	− 0.34	4.07*	0.96 (0.93–1.00)	58.5 (11.4, 32–82)	0.02	0.47	1.02 (0.96– 1.09)
Education	Primary	34 (21.8%)	n.a	n.a	23 (20.3%)	n.a	n.a	n.a	8 (40%)	n.a	n.a	n.a
	Secondary	82 (54.1%)			54 (47.8%)				10 (50%)			
	Tertiary	39 (25.1%)			36 (31.9%)				2 (10%)			
Marital status (yes/no)		123/33	n.a	n.a	26/89	n.a	n.a	n.a	4/16	n.a	n.a	n.a
Time since diagnosis (mean months (SD))		33.9 (15.9)	n.a	n.a	32.3 (16.3)	n.a	n.a	n.a	31.1 (16.7)	n.a	n.a	n.a
Medical treatment satisfaction (0–4 (SD))		3.5 (0.7)	n.a	n.a	3.3 (0.6)	n.a	n.a	n.a	3.1 (1.0)	n.a	n.a	n.a
Ability to handle daily activities well (1–5 (SD))		1.6 (0.7)	7.2	.027	2.1 (0.8)	0.55	5.55*	1.74 (1.10–2.76	2.5 (0.9)	0.56	5.64*	1.75 (1.10–2.78)
Recent life event (yes/no)		109/48	3.9	.140	62/52	0.53	2.55	1.70 (0.89–3.28)	13/7	− 0.28	0.17	0.76 (0.20–2.86)
Previous psychological counselling (yes/no)		121/34	3.3	.189	64/52	0.21	0.32	1.23 (0.60–2.54)	6/14	1.31	3.12	3.72 (0.87–15.97)
Social support (SD)		39.1 (7.4)	19.3	< .001	46.5 (12.1)	0.06	15.67***	1.07 (1.03–1.10)	56.5 (18.4)	0.09	14.73***	1.10 (1.05–1.15)
Optimism (SD)		27.4 (4.2)	8.0	.018	23.7 (4.1)	− 0.10	4.41*	0.91 (0.83–0.99)	19.5 (5.1)	− 0.22	6.40*	0.81 (0.68–0.95)
Neuroticism (SD)		18.6 (4.7)	22.4	< .001	24.2 (4.6)	0.16	15.91***	1.18 (1.09–1.28)	28.2 (4.6)	0.28	12.14***	1.32 (1.13–1.55)

^*^p ≤ .05, **p ≤ .01, ***p ≤ .001

^a^Standard deviation

In univariate analyses, a difference between trajectories was observed for age (*p* = 0.041), previous psychological counselling (*p* ≤ 0.001), perceived social support discrepancy (*p* ≤ 0.001), optimism (*p* ≤ 0.001), neuroticism (*p* ≤ 0.001), experience of a recent life event (*p* < 0.040), and being able to handle daily activities (*p* = 0.037).

The seven significant predictors were entered simultaneously in the multinominal logistic regression analysis with the trajectory no MD symptoms as the reference group (Table 2). The final model was statistically significant (*Χ*^2^ = 166.9, *df* = 14, *p* ≤ 0.001, Cox & Snell R^2^ = 0.45, Nagelkerke = 0.54, McFadden = 0.34). Experiencing a recent life event and previous psychological counselling did not contribute significantly to the overall statistical model (Table 2). Compared to BCS in the reference group, BCS in the trajectories stable&fluc-AD symptoms and any other MD symptoms were less able to handle daily activities, perceived a larger social support discrepancy, and showed less optimism and more neuroticism. Additionally, BCS in the trajectory stable&fluc-AD symptoms were younger compared to BCS in the reference group.

### Trajectories based on LCGA

Using LCGA for the complete sample (*N* = 459), the intercept of the HADS total was 7.6 (95% confidence interval [CI] 7.0–8.1, *p* ≤ 0.001), which can be interpreted as no MD symptoms at baseline. There was a non-significant slope (0.04; 95% CI − 0.11 to 0.19, *p* = 0.630), which can be interpreted as a stable HADS total during 1 year. The most appropriate choice based on fit indices, internal reliability, and interpretability was a three-class model (Table 3). The first trajectory consisted of 269 BCS (58.6%) and was defined as “stable no MD symptoms” (low), as participants reported low baseline HADS total scores (intercept 3.60; 95% CI 3.09–4.11) with a non-significant slope (− 0.09 (95% CI − 0.26–0.07)). The second trajectory was defined as “stable AD symptoms” (AD symptoms). For this trajectory of 151 BCS (32.9%), the intercept was 11.38 (95% CI 10.23–12.54) with a non-significant slope (− 0.1; 95% CI − 0.39–0.19). The third trajectory was defined as “high increasing any other MD symptoms” (high increasing). For this trajectory of 39 BCS (8.5%), the intercept was 19.83 (95% CI 17.54–22.12) with a significantly increasing slope (1.29; 95% CI 0.30–2.29).

**Table 3:** Fit indices, entropy, and average posterior probabilities across models with different number of classes by HADS^a^ score

No. of classes	BIC^b^	LMR-LRT^c^	BLR-T^d^	Entropy	*n*	Posterior probabilities	Intercept (95% CI^e^)	Slope linear (95%CI)
2	9684.567	0.0048	0.0000	0.885	350 (76.3%)	0.968	4.79 (4.02–5.56)***	− 0.06 (− 0.22–0.09)
					109 (23.7%)	0.953	15.68 (13.81–17.55)***	0.29 (− 0.25–0.82)
3	9328.060	0.0001	0.0000	0.880	269 (58.6%)	0.960	3.60 (3.09–4.11)***	− 0.09 (− 0.26–0.07)
					151 (32.9%)	0.918	11.38 (10.23–12.54)***	− 0.10 (− 0.39–0.19)
					39 (8.5%)	0.938	19.83 (17.54–22.12)***	1.29 (0.30–2.29)*
4	9212.377	0.0200	0.0000	0.823	201 (43.8%)	0.916	2.59 (2–3.18)***	− 0.07 (− 0.22–0.08)
					75 (16.3%)	0.903	14.04 (12.74–15.33)***	− 0.14 (− 0.7–0.42)
					32 (7.0%)	0.951	20.50 (18.12–22.88)***	1.48 (0.36–2.6)**
					151 (33.0%)	0.847	7.90 (6.7–9.1)***	− 0.03 (− 0.37–0.31)
5	9201.561	0.3348	0.0000	0.761	158 (34.4%)	0.892	2.09 (1.66–2.51)***	− 0.07 (− 0.21–0.07)
					48 (10.4%)	0.864	15.35 (13.69–17.01)***	− 0.26 (− 0.87–0.35)
					31 (6.71%)	0.942	20.52 (18.04–23.01)***	1.53 (0.36–2.7)***
					133 (29.0%)	0.774	6.13 (5.09–7.17)***	− 0.02 (− 0.4–0.35)
					89 (19.4%)	0.784	10.45 (8.23–12.68)***	− 0.05 (− 0.61–0.51)

^*^p ≤ .05, **p ≤ .01, ***p ≤ .001

^a^Hospital Anxiety and Depression Scale

^b^Bayesian Information Criterion

^c^Vuong-Lo-Mendell Rubin Likelihood Ratio Test

^d^Bootstrap Likelihood Ratio Test

^e^Confidence interval

### Predictors of trajectories based on LCGA

Univariate analyses (Table 4) showed differences between trajectories for age (*p* = 0.015), satisfaction with medical treatment (*p* < 0.004), being able to handle daily activities (*p* < 0.003), previous psychological counselling (*p* ≤ 0.001), experiencing a recent life event (*p* = 0.010), social support discrepancy (*p* ≤ 0.001), optimism (*p* ≤ 0.001) and neuroticism (*p* ≤ 0.001).

**Table 4:** Trajectories based on LCGA^a^: Characteristics & multinominal regression analysis (AD symptoms trajectory and high increasing trajectory^b^ versus low trajectory^c^)

		**Low trajectory**			**AD symptoms trajectory**				**High increasing trajectory**			
**Predictor**			***Χ***^**2**^	***p***		***B***	**Wald**	**Exp (B) (95% CI)**		**B**	**Wald**	**Exp (B) (95% CI)**
Intercept		n.a	8.4	0.015	n.a	− 3.36	3.13	n.a	n.a	− 8.87	7.78**	n.a
Age (mean years (SD^d^; range)		57.8 (9.4; 33–87)	8.3	0.016	55.1 (9.6; 33–78)	− 0.04	7.96***	0.96 (0.93–0.99)	55.5 (10.9; 32–82)	− 0.03	1.67	0.97 (0.92–1.02)
Education	Primary	46	n.a	n.a	31	n.a	n.a	n.a	12	n.a	n.a	n.a
	Secondary	136			69				19			
	Tertiary	84			47				7			
Marital status (yes/no)		214/54	n.a	n.a	119/30	n.a	n.a	n.a	33/6	n.a	n.a	n.a
Time since diagnosis (mean months (SD))		34.0 (15.7)	n.a	n.a	32.8 (16.2)	n.a	n.a	n.a	30.8 (16.8)	n.a	n.a	n.a
Medical treatment satisfaction (0–4 (SD))		3.5 (4.7)	5.0	0.08	3.2 (0.6)	− 0.43	4.24*	0.65 (0.43–0.98)	3.1 (0.9)	− 0.06	0.03	0.94 (0.46–1.93)
Ability to handle daily activities well (1–5 (SD))		1.6 (0.7)	18.8	< .001	2.1 (0.7)	0.63	10.53***	1.87 (1.28–2.73)	2.5 (0.9)	1.24	15.99***	3.46 (1.88–6.37)
Previous life event (yes/no)		87/180	1.9	0.382	70/78	0.31	1.26	1.36 (0.79–2.35)	17/22	− 0.12	0.06	0.89 (0.34–2.35)
Previous psychological counselling (yes/no)		69/198	0.9	0.642	74/77	0.25	0.72	1.29 (0.72–2.31)	24/15	0.00	0.00	1.00 (0.36–2.82)
Social support (SD)		39.7 (8.5)	24.6	< .001	46.8 (12.1)	0.06	16.91***	1.06 (1.03–1.08)	54.8 (16.3)	0.08	18.91***	1.09 (1.05–1.13)
Optimism (SD)		27.4 (4.2)	13.1	0.001	23.9 (4.1)	− 0.07	3.27	0.93 (0.87–1.01)	20.1 (4.6)	− 0.22	11.99***	0.81 (0.71–0.91)
Neuroticism (SD)		18.6 (4.6)	56.7	< .001	24.3 (4.5)	0.21	35.89***	1.24 (1.15–1.32)	28.3 (4.9)	0.34	32.23***	1.40 (1.25–1.57)

^*^p ≤ .05, **p ≤ .01, ***p ≤ .001

^a^Latent class growth analysis

^b^High increasing any other MD symptoms

^c^No MD symptoms

^d^Standard deviation

These eight predictors were included in the final model with the low trajectory as reference group. The final model was statistically significant (*χ*^2^ = 264.5, *df* = 16, *p* ≤ 0.001, Cox & Snell R^2^ = 0.46, Nagelkerke = 0.55, McFadden = 0.35). Age, ability to handle daily activities, social support discrepancy, neuroticism, and optimism contributed significantly to the statistical model. BCS in the AD symptoms trajectory and the high increasing trajectory were less able to handle daily activities, perceived a larger social support discrepancy, and showed more neuroticism compared to BCS in the low trajectory. BCS in the AD symptoms trajectory were younger compared to BCS in the low trajectory, and BCS in the high increasing trajectory reported less optimism compared to BCS in the low trajectory.

## Discussion

In this study, three distinct 1-year trajectories in HADS scores were found in BCS using two different statistical approaches: one approach with clinical cut-off scores to indicate AD symptoms or MD symptoms and one data-driven approach to predict classes of BCS with a similar course of AD symptoms or MD symptoms. The “low” trajectory was found in more than half of the BCS. The second trajectory with (fluctuating) AD symptoms was found in about one-third of the BCS. The trajectory with (high increasing) any other MD symptoms was found in fewer than one in ten BCS. Furthermore, the approach based on cut-off scores showed a very low (1.4%) percentage of BCS with stable AD symptoms and fluctuating scores below and above cut-off scores in almost 40% of the participants. With the latent modelling approach, we found a trajectory AD symptoms in one-third of the BCS, with a wide confidence interval of HADS scores per assessment and no significant change over time. Thus, both statistical approaches showed that AD symptoms can fluctuate in a significant proportion of BCS over time and that a pattern of stable AD symptoms was not present in this sample. This questions the validity of the diagnosis AD in BCS.

The detection of the low trajectories and (high increasing) any other MD symptoms is in line with previous trajectory studies [17, 18, 22, 26, 27]. These studies all reported trajectories as “resilient” and “chronic”, with a stable course of few and high symptoms post-treatment up to 6 months [18, 22, 27], 8 months [26], and 4 years [17]. Non-stable trajectories were observed in all previous studies as well. Our study provided additional detailed observations by means of multiple assessments within a 1-year period, indicating more individual fluctuations in AD symptoms than was expected based on earlier findings. Fluctuations in AD symptoms were found independent of time since diagnosis, which is not in line with the DSM-V definition of AD [3] which assumes that AD diminishes over time, implying a self-healing process [11]. The discrepancies between this study and the established AD criteria stress the debate of AD diminishing after 6 months after AD symptom occurrence or becoming persistent in case of ongoing stressors in the cancer survivor context. Therefore, future research could be directed towards exploring acute and persistent AD immediately post-diagnosis and whether symptoms might fluctuate over time.

Compared to BCS in the trajectories with no MD symptoms, characteristics of BCS in the trajectories of AD symptoms or MD symptoms were a larger social support discrepancy, less optimism, and more neuroticism. These findings are in line with previous trajectory studies, where less social support [17, 27], less optimism [18, 26], and higher scores on neuroticism [18, 26] were observed in “chronic distress” or “lower mental functioning” trajectories. Lower ability to handle daily activities is in line to the criteria of AD [3], where poorer functioning in social relations, work, or study is observed in people who are diagnosed with AD. Lastly, with the exception of Lam et al. [26], previous trajectory studies did not find age differences between trajectories, which is contradictory to our study. BCS with a trajectory of (fluctuating) AD symptoms were almost 3 years younger compared to BCS belonging to the trajectory no MD symptoms. A systematic review including cross-sectional and longitudinal studies found that a younger age increased the risk of distress [40]. These previous findings regarding predictors combined with the results of our study emphasize the relevance for clinicians to monitor these predictors to detect vulnerable BCS showing AD symptoms.

### Study limitations

The results should be interpreted carefully because of selection bias in the study sample. Participants who completed all questionnaires were older and lower educated compared to participants who did not complete all questionnaires, although the sample used for the cut-off score analysis was comparable to the sample in the LCGA analysis. Furthermore, analysis of the predictive value of education was not possible due to too small cells, resulting in inconclusive findings on education. In our study, we assessed whether participants had previously received psychological counselling. We did not, however, assess whether participants had a history of mental illness, which could have been an important predictor to developing AD. This study was an additional analysis of a dataset on the course of fear of cancer recurrence (FCR) over time in BCS [15]. Secondary analyses reduce research participation burden, but results might be less generalizable to the overall BCS population, since BCS signed up for research investigating FCR instead of AD related to cancer.

For research purposes, the analyses of the HADS are of great value to gain insight in which BCS are at risk for AD. The HADS, however, does not assess impairments in social or occupational functioning, which is a limitation. While not assessed thoroughly in our study, BCS with a trajectory of (fluctuating) AD symptoms reported less ability to handle daily activities and had a larger perceived social support discrepancy. A diagnostic interview, use of an AD-specific questionnaire, or combining measures would capture AD more accurately. Finally, due to the small number of participants with AD symptoms, we were not able to further categorize subtypes of AD.

### Clinical implications

This study used two different approaches to analyse the data, combining methodologies used in previous studies to observe the course of AD symptoms: a clinical point of view in using a cut-off score to screen for a possible AD or MD and a statistical point of view to predict latent classes based on scores over time. Both methodologies detected fluctuating symptoms over time. This would imply that conclusions based on single assessment HADS scores in clinical practice would not be sensitive enough to detect those patients with AD symptoms and for whom a diagnosis of AD might be applicable.

## Conclusion

A substantial proportion of BCS up to 5 years post-diagnosis showed fluctuating AD symptoms, and only a negligible percentage of the cases had a stable course of AD symptoms. We suggest handling single assessment cut-off scores with caution.

## Data Availability

Data are available upon reasonable request. Requests to access the datasets should be directed to jose.custers@radboudumc.nl.

## Declarations

### Disclaimer

The study sponsor had no role in the study design, data collection, analysis and interpretation of data, writing of the report, or decision to submit the paper for publication.
